# Supplementary material for: The genome sequence of the Purple Cloud, Actinotia polyodon (Clerck, 1759) (Lepidoptera: Noctuidae)
Source: Wellcome Open Res. 2026 Jan 16;11:40. [Version 1] doi: 10.12688/wellcomeopenres.25772.1 (PMC12902673; doi:10.12688/wellcomeopenres.25772.1)
Supplement: Peer review history

# Reviewer report: Reviewer response for version 1

**DOI:** 10.21956/wellcomeopenres.28373.r145390

**Published:** 2026-02-17

**Authors:** Ariel Chipman

**Affiliations:** 1 Hebrew University of Jerusalem School of Pharmacy, Jerusalem, Israel

The authors assembled the genome of the noctuid moth Actinotia polyodon from a single female collected in France. Sequencing and assembly were done according to well established protocols, by a research group with ample experience in using these protocols. The quality of the assembled genome is very high, with chromosome-length scaffolds corresponding to the expected number of chromosomes, including the W and Z chromosomes, as wells the mitochondria as a separate scaffold. The work adheres to expected standards and is presented as is customary in the field. I have no comments regarding the manuscript.

Are sufficient details of methods and materials provided to allow replication by others?

Yes

Is the rationale for creating the dataset(s) clearly described?

Yes

Are the datasets clearly presented in a useable and accessible format?

Yes

Are the protocols appropriate and is the work technically sound?

Yes

Reviewer Expertise:

Arthropod evolution

I confirm that I have read this submission and believe that I have an appropriate level of expertise to confirm that it is of an acceptable scientific standard.

# Reviewer report: Reviewer response for version 1

**DOI:** 10.21956/wellcomeopenres.28373.r145970

**Published:** 2026-02-12

**Authors:** Jonathan Badger

**Affiliations:** 1 National Cancer Institute, Bethesda, Maryland, USA

First of all, please check the author list to make sure it is correct -- currently the first two authors are listed as oreina, Papillons de France. I understand that this butterfly organization contributed to this work, but please recheck the authorlist.

 Other than that, I think this is a well written genome note that gives sufficient detail as to background, methods, and results. I particularly liked the inclusion of Table 5, as details of the software used are often missing in these data notes. 

 You may want to include some discussion of why you had issues with the ordering of contigs on chromosome 28 and not the other ones.

Are sufficient details of methods and materials provided to allow replication by others?

Yes

Is the rationale for creating the dataset(s) clearly described?

Yes

Are the datasets clearly presented in a useable and accessible format?

Yes

Are the protocols appropriate and is the work technically sound?

Yes

Reviewer Expertise:

I am a computational genomicist primarily working in biomedical research, although I have also contributed to insect, fungal, and protist genome projects.

I confirm that I have read this submission and believe that I have an appropriate level of expertise to confirm that it is of an acceptable scientific standard.

# Reviewer report: Reviewer response for version 1

**DOI:** 10.21956/wellcomeopenres.28373.r145969

**Published:** 2026-02-06

**Authors:** Kathy Darragh

**Affiliations:** 1 Indiana University Bloomington, Bloomington, Indiana, USA

This is a standard genome note. All methods are fully described with sufficient background on the species and a specimen photograph. All information is clear and well presented. Data has been made available and this is also included in the note. This genome will contribute to comparative genomics projects. I have no suggestions for the authors.

Are sufficient details of methods and materials provided to allow replication by others?

Yes

Is the rationale for creating the dataset(s) clearly described?

Yes

Are the datasets clearly presented in a useable and accessible format?

Yes

Are the protocols appropriate and is the work technically sound?

Yes

Reviewer Expertise:

Evolutionary biology, genetics.

I confirm that I have read this submission and believe that I have an appropriate level of expertise to confirm that it is of an acceptable scientific standard.
